# The Hospitals of Athens

**Published:** 1900-10-13

**Authors:** 


### The Institutional Workshop.

### THE HOSPITALS OF ATHENS.

Modern Athens, with its historical interest and the romance which attaches to it, is rather a depressing place to visit, in view of the sad havoc which has been made with the ancient buildings and relics by various despoilers of more than one nationality, who have deprived its temples and ruins of some of their most treasured architectural features. Still, modern Athens is a beautiful city, and it is to be noticed that many of the residents are as devoted to athletics and outdoor pursuits as we are at home.

Before the war with Turkey, the hospitals in Greece were very unsatisfactory in many respects. On the outbreak of the war the Queen and Royal Princesses threw themselves heart and soul into the work of renovation, and determined that no effort of theirs should be wanting to make the hospital provision for the Greek people efficient and as near the standard of modern requirements as possible. Having set their hands to the work, they have continued to labour at it ever since. When we recently made an inspection of the three chief hospitals in Athens—i.e. the Queen's Hospital, Princess Sophie's Hospital for Children, and the Military Hospital—we were agreeably surprised to find that each of these establishments was in excellent order, and that the general appearance and up-keep were surprisingly good. The credit of this great improvement in the hospitals of Athens is mainly, if not entirely, due to the Royal Family of Greece. Unfortunately, they have been hampered by want of funds, but, despite this drawback, by the exercise of great liberality and continuous effort they have accomplished so much that they deserve and should receive the most liberal support of every member of the Greek nation, no matter where he may reside. We are of opinion that it would redound to the lasting credit of the Greek community in Great Britain, many of whom have the reputation of being very wealthy, if they were to make up a purse of, say, £10,000, and send it to the Queen of Greece at Christmas, with authority to expend it upon the provision of hospital and medical comforts for the people of Athens, at the discretion, say, of Her Majesty and the Princess Sophie.

### The Queen's Hospital.

This institution is the special care and under the immediate direction of the Queen of Greece. Her Majesty is a frequent visitor: she goes to the hospital constantly, without any previous warning, and to her energy and initiative are due the very remarkable reforms which have been introduced into its administration during the last few years. Everywhere throughout the establishment evidence is to be found of the practical effects of the Queen's influence and interest. Considering the buildings are not new, and that they were built without reference to several sound principles which govern hospital construction at the present day, we were very greatly impressed at the high average of efficiency which prevailed throughout every part of the establishment. The patients were evidently comfortable; the staff were zealous and anxious to promote in every way the comfort and recovery of the patients; and the furniture and fittings, the improved operation theatre, its appurtenaces, and many other matters, all tended to demonstrate how thoroughly the Queen has studied hospital matters, and how great has been her influence for good in the city of Athens.

### Princess Sophie's Hospital for Children.

This hospital is a new institution erected in Athens entirely by the energy and liberality of the Crown Princess of Greece. It is planned upon the pavilion principle, and the arrangements of the various blocks devoted to the different diseases to which children are specially liable have been well thought out, whilst the details of construction and the materials used leave little to desire. At the time of our visit the first pavilion and the administration block had just been completed; the wards were cheerful, and, in regard to decoration, furniture and fittings, were admirably conceived and organised. The nursing was under the direction of an English lady, who is also a trained nurse; and the whole appearance of the hospital was so tasteful and attractive, so far in advance of anything yet attempted in the way of hospital accommodation in this part of Europe, that critics are not wanting to state that this very excellence amounts to extravagance. Such a charge is not only ungracious, but ridiculous, and it may be some consolation to all who are interested in the improvement of the hospitals of Greece to know that theirs is not the only country where reform in hospital administration and the introduction of perfect cleanliness and orderliness throughout establishments of the kind have been resented on the ground of extravagance.

Great difficulty is experienced in securing the right type of nurse for hospitals in Greece, and we gathered that the experience of those interested in hospitals there is identical with the experience of those at home who actively concerned themselves at the outset with the provision of nurses for our army in South Africa. The truth is, and it may perhaps be usefully stated, that those who are most insistent upon being selected for posts of this kind are often ill adapted to the work and lack suitable training and experience. Evidence of better training and less emotion have been wanting to a large extent, the knowledge of which facts may, no doubt, effect a remedy in due course.

We hope to publish the plans of this hospital later on. It is beautifully situated near the golf ground: each pavilion has pleasant balconies, ample accommodation for the open-air treatment, and Princess Sophie is to be heartily congratulated upon the successful achievement of a difficult task, which reflects the greatest honour upon herself and all who have co-operated with her in this good and necessary work.

### The Military Hospital.

The Military Hospital is a large building with wide corridors and large, airy, well-lighted wards. It contains some hundreds of beds. Thanks to the energy of the members of the Royal Family, it has recently been brought up to a state of efficiency which makes it a pleasant establishment to visit, and proves what can be done with very imperfect materials when there is a will to set about doing it in the best attainable manner. The whole of the interior of the hospital has been redecorated most tastefully; the floors are good, and the fittings are gradually being brought up to modern requirements. In appearance it presented a favourable contrast to some of our own military hospitals at home, and the patients seemed perfectly happy and well cared for. Much more would be done if money were forthcoming, but with the limited resources available it was surprising to find the high standard of efficiency in many directions which has already been attained.

A Tribute to Royalty.

We spent nearly the whole of one day in the hospitals of Athens. Everywhere the influence of the Royal Family was exhibited by the marked improvements introduced within the last two or three years. The Queen of Greece devotes her special attention to the Queen's Hospital, which she visits two or three times a week, and over which she exercises a most beneficent influence, to the great comfort of the patients and the increased efficiency of the whole establishment. Both the doctors and the staff were loud in their praise of the splendid devotion of the Queen to the cause of her suffering subjects, and her good influence is everywhere apparent, for Her Majesty makes it her business to see that every detail of the hospital is carefully looked into, and admirable economy and method have so been secured. It was interesting to note that there are pay wards and free wards in the Queen's Hospital, and that in Greece the population recognise that it is a privilege to be allowed to pay according to their means for the expense of their treatment while inmates of a hospital.

The Crown Princess and Princess Marie have chiefly interested themselves in the Children's Hospital and the Military Hospital respectively, and the condition of both these institutions shows how much good royal personages can accomplish under difficult circumstances when they put their heart into the work and are prepared to be prodigal of the time they give to important efforts of this character. The people of Greece owe much to the ladies of the reigning house, and the result of our inspection gave rise to a feeling of real pleasure and satisfaction that the Queen and the two Princesses should have been able to accomplish so much for the Greek people in the way of hospital care and efficiency, despite the many difficulties and discouragements which they have to face owing to circumstances which, however natural, are none the less very difficult to overcome without exciting prejudice or leading to ultimate defeat. Whatever may be said against our modern methods of civilisation, it is at least true that in the present day the reigning families of Europe, and especially all who are related to the Royal Family of England, by their attention and the time they devote so usefully to philanthropic objects, and especially to the care of the sick, set an example to all classes of their subjects, which is stimulating public interest and promoting efficiency of administration to an extent greater than most people at present appreciate.

